# Factors Related to the Willingness of People with Mental Health Illnesses Living in Group Homes to Disclose Their Illness to Supporters during Disaster Evacuation: A Cross-Sectional Study

**DOI:** 10.3390/nursrep14020076

**Published:** 2024-04-22

**Authors:** Masato Oe, Hisao Nakai, Yutaka Nagayama

**Affiliations:** 1 Nursing Department, School of Nursing, Kanazawa Medical University, Kanazawa Medical University Hospital, 1-1 Uchinada, Kahoku 920-0265, Japan; oemasato@kanazawa-med.ac.jp (M.O.); naga-y@kanazawa-med.ac.jp (Y.N.); 2 Faculty of Nursing, University of Kochi, 2751-1 Ike, Kochi 781-8515, Japan

**Keywords:** natural disasters, mental disorders, group homes, emergency shelter

## Abstract

Severe heavy rains caused by linear precipitation systems are occurring more frequently in Japan owing to climate change, and residents are being asked to evacuate more often. The purpose of this study was to identify factors associated with the willingness of people with mental health illness (PMHI) in group homes to disclose their illness when being evacuated. Participants were PMHI living in group homes in Japan. We conducted an original anonymous self-administered questionnaire based on previous research. Valid data from 119 people were analyzed. Factors associated with the willingness to disclose illness to supporters upon evacuation were “I can imagine living in a public shelter” (Odds Ratio [OR] 4.50, 95% Confidence Interval [CI]: 1.78–11.43), and “I socialize with neighbors” (OR 5.63, 95% CI: 1.74–18.22). Managers of group homes should encourage PMHI to imagine life in an evacuation zone by increasing opportunities for disaster training and for interaction with local residents. People who are less likely to socialize with neighbors should be especially careful, as they may not be able to disclose their illness, and those who support evacuees should pay special attention to these people.

## 1. Introduction

Group homes are residences where long-term hospitalized patients can undergo life training before being discharged and living in the community. Target group home users wish to live alone but are often unsure about living in the community immediately after hospital discharge [1]. The number of group homes for people with mental health illness (PMHI) in Japan is increasing; as of February 2021, there were approximately 140,000 group home residents [2]. Although the increase in group homes has contributed to the deinstitutionalization of psychiatric hospitals, managing PMHI who do not wish to move out of group homes and those who cannot move out because they are older and require nursing care remains challenging [3,4].

In recent years, climate change has exacerbated many large-scale disasters such as heat waves, wildfires, and heavy rain caused by linear precipitation systems [5,6]. People who are undergoing treatment for psychiatric problems are more vulnerable to unfamiliar environments and stress. Secondary stressors and previous psychiatric problems can have detrimental effects on mental health following disasters [7]. Moreover, there is evidence of a relationship between the number of times a disaster victim experiences secondary stress, such as the death of a loved one or the destruction of property, and the subsequent development of mental disorders [8]. Exposure to secondary stress is an important issue because it can impair the mental health of disaster victims and, in the worst cases, lead to the development of psychosis.

PMHI in different societies and cultures are exposed to prejudice and discrimination [9], and may even experience stigma during disaster evacuations [10]. For example, a study of individuals being treated for drug dependence identified prejudice and discrimination from medical professionals and health personnel that affected treatment and disaster evacuation [11]. Stigma prevents PMHI from seeking professional consultation and medical treatment after a natural disaster, and is a barrier to the provision of sociopsychological support [12]. Living in an evacuation shelter also has a substantial negative effect on mental health. Behavioral and mental health issues accounted for 7% of the reasons given for visiting evacuation shelters to seek volunteer Red Cross disaster health services during Hurricane Harvey in 2017. Of the 7% of people with behavioral and mental health issues, approximately half experienced behavioral or mental health symptoms and 20% expressed agitation or disruptive behavior [13]. Even after safe evacuation after the Fukushima Daiichi Nuclear Power Plant accident caused by the Great East Japan Earthquake in 2011, some psychiatric patients were not comfortable living in groups. According to one hospital evacuation report, 10 of the 48 psychiatric patients evacuated to a shelter in Fukushima were unable to adapt to the shelter environment and left the shelter [14]. Therefore, although evacuation greatly reduces casualties, there is substantial evidence that living in evacuation centers has serious physical and mental health effects [15]. This suggests that environmental changes and exposure to stress in evacuation shelters can be difficult to cope with, especially for people receiving treatment for mental illness or mental health issues.

Research following the 2011 Great East Japan Earthquake showed that strong social support can help people to develop psychological resilience to disasters [16]. To reduce the negative mental health effects of disasters, it is important to identify psychosocial problems, symptoms, levels of functioning, attitudes, beliefs, and the status of existing mental illnesses in survivors [17]. However, unlike people who have been physically injured by disasters, the disabilities of people with chronic illnesses are invisible [18]. Therefore, during disasters, it is important that PMHI inform supporters of their symptoms to ensure that they receive appropriate social support. However, there are no studies on the effect of self-disclosure on the experiences of PMHI during disasters.

The implementation of the Community-Based Integrated Care System for Mental health disability by the Japanese Ministry of Health, Labour and Welfare will facilitate the move of more psychiatric patients from hospitals to the community and will increase the number of group homes [19]. Considering the increasing risk of disasters caused by climate change, as evidenced in recent years [20,21], it is important to improve the disaster preparedness and evacuation response of PMHI in group homes. The purpose of this study was to identify predictors of the willingness of PMHI living in group homes in Japan to disclose their illness to supporters during disaster evacuation. The findings will help to identify PMHI in group homes in Japan who do not disclose their illness to others during evacuations, provide details of their evacuation behavior, and offer measures for assessing their evacuation experiences during disasters.

## 2. Materials and Methods

### 2.1. Research Location

**Figure 1:**
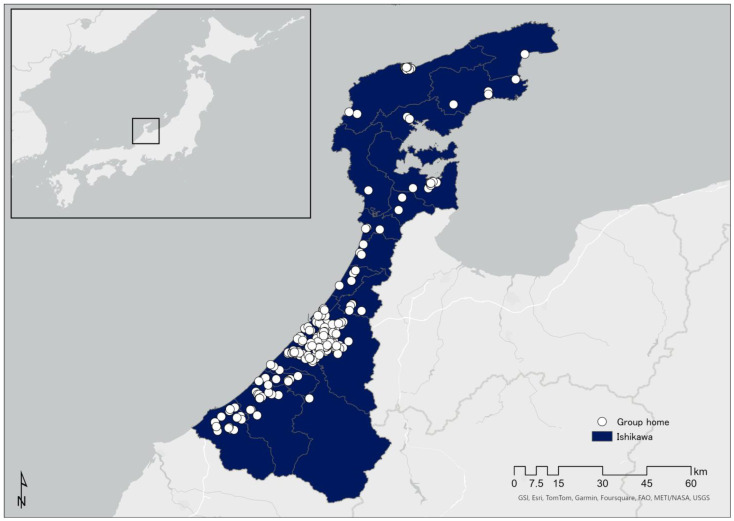
Location of Ishikawa Prefecture and geographical distribution of group homes.

The research location was Ishikawa Prefecture, which faces the Sea of Japan (see Figure 1). Located in the Hokuriku region (Toyama, Ishikawa, Fukui), it is long and narrow from southwest to northeast and comprises approximately 4186 square kilometers [22]. As of July 2021, the population was 1,127,428, of whom 18,307 (3.6% of the national total) suffer from mental illness [23].

### 2.2. Data Collection

The participants were PMHI who use a group home for mentally ill people in Ishikawa Prefecture, Japan. The selection criteria were people who had been diagnosed with a mental illness and were living in a group home. We asked 306 group homes published on the Ishikawa Prefecture website to complete a web-based questionnaire survey [24]. For those who did not have access to the web-based survey, we distributed a paper survey and asked them to complete it. We created an original, anonymous, self-administered questionnaire based on a previous survey of the post-earthquake living conditions of people with mental disabilities. The previous survey investigated the actual living conditions of mentally disabled people in Minamisoma City who were victims of the 2011 Great East Japan Earthquake and possessed mental disability certificates [25]. Additionally, we referred to the website of the Japan Broadcasting Corporation, which provides information useful for people with disabilities in preparing for, and in the event of, a disaster [26]. With reference to these sources, we developed a candidate list of questions related to PMHI evacuation actions and support during disasters. We held an expert committee comprising psychiatric nurses, visiting nurses, and public health nurses, as well as researchers in psychiatric nursing, home nursing, and disaster nursing, to discuss the list of candidate questions. The items were revised to improve the understandability, relevance, and validity of the questionnaire. A pre-test was then conducted with several nursing researchers, and some items were revised based on the results.

We generated our survey using SurveyMonkey, a cloud-based survey development application. The original questionnaire was distributed by mail, explaining the purpose and significance of the study, and the survey method, and reassuring respondents that participation was voluntary, their responses were anonymous, and individuals would not be identified by completing the questionnaire.

This study was conducted from 15 December 2022 to 27 January 2023. This manuscript was drafted based on the Strengthening the Reporting of Observational Studies in Epidemiology (STROBE) guidelines for cross-sectional studies [27].

### 2.3. Survey Contents

#### 2.3.1. Participant Background Information

Sex, age, and type of disability (mental disability, intellectual disability, and physical disability).

#### 2.3.2. Services Used by Participating PMHI

Day services, visiting services, employment support services, other services.

#### 2.3.3. Where Participants Lived before Hospitalization

Hospital, home, other group homes, other places.

#### 2.3.4. Where Participants Obtained Information to Make Evacuation Decisions

Smartphone, feature phone, computer, TV, radio, group home staff, local government staff.

#### 2.3.5. Participants’ Typical Socializing Patterns

Socialize with group home PMHI, socialize with neighbors; the response options were: “I don’t do it”, “I don’t do it much”, “I do it a little”, and “I do it regularly”.

#### 2.3.6. Assumptions about Evacuation from a Disaster

My friends would support me if I had to evacuate; My family would support me if I had to evacuate; My group home staff would support me if I had to evacuate; I can imagine living in a public shelter; I want to stay in my room without evacuating; I can’t live in a shelter with many people; I am concerned about interpersonal relationships at the shelter; I am concerned about stigma from others at the shelter; I disclosed my illness to supporters after the evacuation. The response options were: “no”, “somewhat no”, “somewhat yes”, and “yes”.

### 2.4. Analysis

Of the participants who answered the questionnaire, 119 provided valid data for all of the items listed above. We calculated the mean age and its standard deviation (SD) to understand the characteristics of the participants. Furthermore, we calculated the age distribution for each 10-year age group. To consider factors associated with the willingness to “disclose my illness to supporters after the evacuation”, age was divided into “older adults” (65 years and older) and “under 65 years”, and type of disability was divided into “mental disability” and “mental disability and other disabilities” (physical disability, intellectual disability). Socializing with people was divided into “No” for “I don’t do it” and “I don’t do it much”, and “Yes” for “I do it a little” and “I do it”. For assumptions about evacuation from a disaster, “no”, and “somewhat no” comprised “No”, and “somewhat yes” and “yes” comprised ”Yes”. Relationships among the responses to the following items were analyzed using the χ^2^ test or Fisher’s exact test: “disclosed my illness to supporters after the evacuation” and PMHI background, mobile device usage, socializing with people, sources of information for determining disaster experience and evacuation, sources of information when deciding whether to evacuate, and assumptions about evacuation from a disaster. To analyze the factors that influenced the willingness of PMHI to disclose their illness to a support person after evacuation, the objective variable was “disclosed my illness to supporters after the evacuation” and the confounders were sex, age group, type of disability, and experience of being affected by a natural disaster. We conducted a binary logistic regression analysis using the following explanatory variables that had a significance probability of less than 5% in the univariate analysis: “I can imagine living in a public shelter” and “I socialize with neighbors”. Each selected variable was forced in after checking multicollinearity (variance inflation factor ≥ 10). The significance level was set at 5%. SPSS version 29 (IBM Corporation, Armonk, NY, USA) was used for all statistical analyses.

### 2.5. Ethical Considerations

This study was conducted with the approval of the University Medical Research Ethics Review Committees at the authors’ universities (No. I765). The participants were given a written informed consent form and were informed of the purpose and importance of the study, the survey method, the fact that participation was voluntary, and the fact that they would not be personally identified when the results were made public. Participants completed a self-administered questionnaire. Completion of the questionnaire implied their consent.

## 3. Results

### 3.1. Characteristics of Participants

Of the 1857 PMHI using 306 group homes in Ishikawa Prefecture, 234 (12.6%) responded: 214 via the paper-based survey and 20 via the web-based survey. The sample retained for analysis was 119 people (6.4%) who answered all of the following items and returned complete data:

“Participant background”, “Services used by PMHI”, “Mobile device usage”, “Sources of information for determining disaster experience and evacuation”, “Sources of information when deciding whether to evacuate”, “Assumptions about evacuation from a disaster”, and “Disclosed my illness to supporters after the evacuation”.

The mean (SD) age of participants was 50.9 years (15.4), with 25 (21.0%) in their 50s, 25 (21.0%) in their 60s, and 20 (16.8%) in their 40s. Regarding the type of disability, 99 (83.2%) reported having a “mental disability” and 20 (16.8%) reported having “other disability”. Of those with “other disability”, 10 (8.4%) reported having both a mental disability and intellectual disability, 7 (5.9%) reported having both a mental disability and physical disability, and 2 (1.7%) reported having an intellectual disability. Regarding the services used by PMHI, 51 (42.9%) used employment support services, 48 (40.3%) used day services, and 39 (32.8%) used visiting services (see Table 1).

### 3.2. Whereabouts before Hospitalization

**Figure 2:**
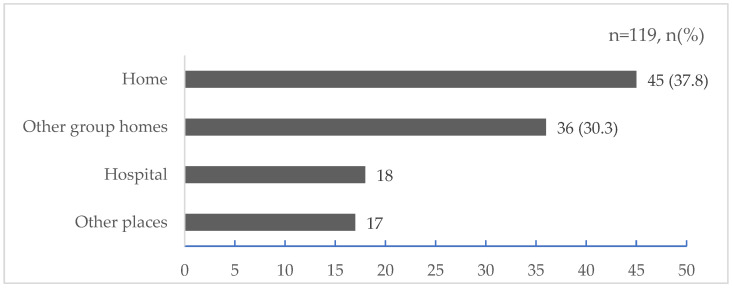
Where participants lived before hospitalization (multiple answers).

Regarding where participants lived before they were hospitalized, 45 (37.8%) said they were at home, 36 (30.3%) said they lived in another group home, and 18 (15.1%) said they lived in a hospital. Details are shown in Figure 2.

### 3.3. Sources of Information for Evacuation Decisions

**Figure 3:**
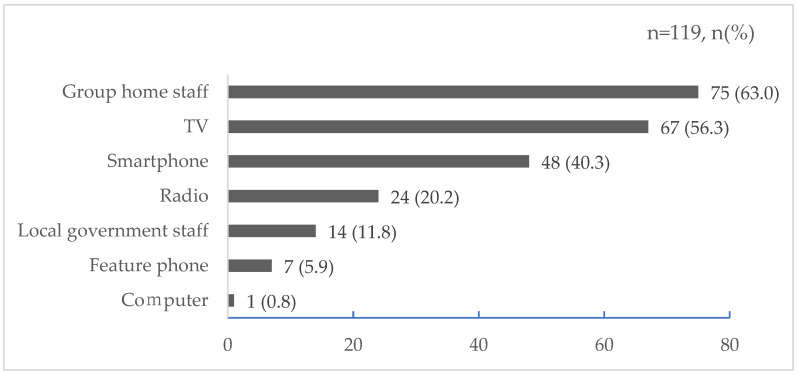
Sources of information for evacuation decisions (multiple answers).

Participants were divided as follows in their responses to sources of information for evacuation decisions: 75 (63.0%) chose group home staff, 67 (56.3%) chose TV, and 48 (40.3%) chose smartphone. Details are shown in Figure 3.

### 3.4. Results of Cross-Tabulation of Responses to Each Item and “Disclosed My Illness to Supporters after the Evacuation”

The results of the univariate analysis using cross-tabulation are shown in Table 2. Among PMHI in group homes, the following variables were significantly associated with the willingness to “disclose my illness to supporters after the evacuation during a disaster”:

“Yes” to “socialize with neighbors” (*n* = 22, 18.5%; *p* = 0.001), and “yes” to “I can imagine living in a public shelter” (*n* = 38, 31.9%; *p* = 0.001).

### 3.5. Binary Logistic Regression Analysis Indicated Factors Associated with the Willingness to “Disclose My Illness to Supporters after Evacuation during a Disaster”

Table 3 shows the results of a binary logistic regression analysis using as the dependent variable the willingness of PMHI in group homes to “disclose my illness to supporters after the evacuation” in the event of a disaster. After controlling for the effects of sex, age group, type of disability, and experience of being affected by a natural disaster, the following factors were associated with the willingness to “disclosed my illness to supporters after the evacuation”: more individuals answered “yes” than “no” for “I can imagine living in a public shelter” (OR 4.50, 95% CI: 1.78–11.43), and more answered “yes” than “no” for “socialize with neighbors” (OR 5.63, 95% CI: 1.74–18.22). Details are shown in Table 3.

## 4. Discussion

In this study, we identified factors associated with disaster preparedness among PMHI living in group homes and their willingness to disclose their illness when evacuated during a natural disaster. Socializing with neighbors and the ability to imagine living in a public shelter were identified as factors associated with the willingness to disclose illness to supporters upon evacuation among PMHI living in group homes.

According to a 2018 nationwide survey of group homes in Japan, 26.1% of PMHI were then in their 50s, 24.9% in their 60s, and 22.7% in their 40s [28]. The distribution of our participants in the present study aligned with their sample and thus likely represents the general population of PMHI living in group homes in Japan. Furthermore, according to that national survey, 36.7% of people lived in a hospital before entering a group home, 35.5% lived at home, 7.8% in another residential facility, and 7.7% in another group home. In recent years, the number of group homes has increased in Japan owing to the deinstitutionalization of psychiatric hospitals [28]. The role of a group home is to provide life skills training to patients who have been discharged from a psychiatric hospital; typically, they are worried about living in the community immediately after their discharge, and their life skills are too immature to allow them to live on their own [29]. According to the national survey mentioned above, the most common place for PMHI to live immediately before entering a group home was a hospital, followed by their home, another facility, or another group home [28]. Therefore, given the recent wave of deinstitutionalization, these groups can be considered generally similar.

In recent years, social media platforms using smartphones have become an increasingly popular way to obtain information during disasters [30,31]. In Japan, disaster-related information has traditionally been provided through television broadcasts after a disaster occurs. In a 2016 survey, television was the most popular source for people’s disaster information [32], but in a 2022 survey, the younger people were, the more likely they were to obtain disaster information from their smartphones [33]. Furthermore, when choosing where to obtain information when deciding whether to evacuate, excluding group home staff who regularly support the daily lives of PMHI, people’s most common source of information was television and then smartphones, which is consistent with recent trends in Japan.

Even taking into account the effects of age, sex, type of disability, and experience of being affected by a natural disaster, we found that PMHI who can imagine life as an evacuee in a public shelter and who engage in socializing with neighbors may be more willing to disclose their illness to supporters during an evacuation.

According to a 2014 general population survey, people who can imagine life in a public evacuation center are able to predict the impact of a disaster on themselves and are more likely to encourage others to evacuate [34]. Conversely, people who cannot imagine what life would be like after evacuation are probably less likely to evacuate. Individuals who do not evacuate will not have the opportunity to decide whether to disclose their illness to a support person. Therefore, the ability to imagine life after evacuation seems important, regardless of any illness disclosure.

In recent years, virtual reality and augmented reality have been used to simulate disaster damage and evacuation life, in addition to training and videos [35]. This may be an effective intervention for PMHI who are hesitant or resistant to participating in disaster training with others.

The Japanese government has published what others must take into consideration when evacuating with PMHI. Specifically, PMHI often have chronic mental illnesses that interfere with their social lives and interpersonal relationships, and they may not be able to adapt to group life at evacuation shelters [36,37]. Therefore, to prevent PMHI from becoming isolated in evacuation shelters, it is necessary to provide accommodations that allow them to live with acquaintances and friends [37]. Importantly, however, the medical conditions and disabilities that PMHI have are often difficult to discern from their appearance, and thus, even if they are in a crisis situation after being affected by a disaster, they may not receive appropriate consideration [38]. Therefore, when evacuating, it is advisable that a person disclose their illness to their supporters and seek appropriate support. Such support should include the provision of specialized mental health care, as well as designated quiet areas or cubicles for individuals who need their own space. Supporters need to prepare supplies and conduct evacuation drills with PMHI residents during normal times in anticipation of future evacuations. However, it is not easy for PMHI to ask supporters for help in the event of a disaster or emergency. It is well known that, traditionally, PMHI have suffered from stigmatization [39,40], and the stigma surrounding mental illness is a barrier to PMHI disclosing their illness and seeking professional help [41,42,43,44]. Indeed, given the many reports confirming that the stigma around PMHI delays access to specialists even in normal circumstances, seeking help from evacuation shelters during disasters can be quite difficult. Thus, our finding that PMHI who closely socialize with their neighbors are more likely to be willing to disclose their illness to supporters in the event of a disaster is of particular importance. It suggests that the impact of stigma on PMHI may be reduced by living in group homes and interacting with the community. It has also been shown that stigma can be reduced by increasing opportunities for interaction between PMHI living in the community and general residents [45]. Direct social contact between PMHI and local people has been cited as one of the effective strategies to reduce stigma [42,46,47]. Therefore, living together in a group home, interacting with others, and getting along with neighbors may contribute to protecting oneself during evacuation in a disaster. However, this association requires further detailed investigation.

This study has some limitations. First, participants in this study represented only 6.4% of PMHI residing in group homes in Ishikawa Prefecture, Japan. Second, the timing of the survey may have influenced the results because it was not conducted during a disaster-prone period. Third, study participants who had only recently started living in a group home may not have had sufficient interaction with other group home users and neighborhood residents. One survey of group homes for PMHI in Tokyo, Japan, found that although approximately 57% of such homes engaged in community exchange, 68% did not disclose that their group home users were PMHI because of concerns about stigma and discrimination [48]. It is also possible that participants who had just started living in a group home may not have thought about disasters and evacuations in that specific context. Finally, we were unable to measure whether stigma toward PMHI was a barrier to their disclosing their illness. Additional studies are needed to determine if PMHI who self-disclose illness information are more likely to stay in a shelter and/or to experience a reduction in negative symptoms while in the shelter. Research is also needed on the effects of stigma on self-disclosure among PMHI. Additionally, longitudinal studies are warranted to investigate what kind of interventions are required from supporters and which interventions are more effective. There is evidence that community-based training, participation in community-based activities, interaction with the wider community, and involvement in disaster simulations can help to minimize the effects of disaster on vulnerable populations and increase community support for these populations [49,50]. We recommend that group home users maintain preparedness for evacuation to a public shelter and engage in training that simulates actual evacuation life. Specifically, this would include not only group home evacuation drills, but also participation in community-based evacuation drills and activities with other community members.

Given these limitations, the results of this study can be generalized only with caution. Finally, this was a cross-sectional study, and thus a causal relationship between the variables under investigation cannot be established.

## 5. Conclusions

For PMHI who live in group homes, being able to imagine life in an evacuation zone and habitually socializing with neighbors may be predictive factors for their willingness to disclose their illness to supporters during an evacuation.

## Figures and Tables

**Table 1:** Relationship between participant background and willingness to disclose an illness to supporters after the evacuation (*n* = 119).

				Disclose My Illness to Supporters after the Evacuation					
Item	Category	Total		No		Yes			
		*n*	%	*n*	%	*n*	%	*p*-Value	
Participant background									
Sex	Male	78	65.5	44	56.4	34	43.6	0.589	a
	Female	41	34.5	21	51.2	20	48.8		
Age, median (SD)	50.9 (15.4)			65	54.6	54	45.4	0.168	a
	10 s	2	1.7	2	100.0	0	0.0		
	20 s	13	10.9	4	30.8	9	69.2		b
	30 s	15	12.6	10	66.7	5	33.3		
	40 s	20	16.8	11	55.0	9	45.0		b
	50 s	31	26.1	13	41.9	8	25.8		
	60 s	25	21.0	15	60.0	10	40.0		a
	70 s	11	9.2	10	90.9	1	9.1		
	80 s	2	1.7	0	0.0	2	100.0		a
Age group	Under 65 years old	92	77.3	46	50.0	46	50.0	0.062	a
	65 years or older	27	22.7	19	70.4	8	29.6		
Type of disability	Mental disability	99	83.2	58	58.6	41	41.4	0.053	a
	Other disabilities	20	16.8	7	35.0	13	65.0		
	Mental disability and intellectual disability	10	8.4						
	Mental disability and physical disability	7	5.9						
	Intellectual disability	2	1.7						
	Mental disability, intellectual disability, and physical disability	1	0.8						
Services used by PMHI (Multiple answers)									
Day services	No	71	59.7	38	53.5	33	46.5	0.769	a
	Yes	48	40.3	27	56.3	21	43.8		
Visiting services	No	80	67.2	46	57.5	34	42.5	0.366	a
	Yes	39	32.8	19	48.7	20	51.3		
Employment support services	No	68	57.1	42	61.8	26	38.2	0.071	a
	Yes	51	42.9	23	45.1	28	54.9		
Other services	No	109	91.6	61	56.0	48	44.0	0.509	b
	Yes	10	8.4	4	40.0	6	60.0		

a: χ^2^ test; b: Fisher’s exact test.

**Table 2:** Relationship between the willingness of PMHI to disclose their illness to supporters after the evacuation and multiple variables (*n* = 119).

				Disclosed My Illness to Supporters after the Evacuation					
Item	Category	Total		No		Yes			
		*n*	%	*n*	%	*n*	%	*p*-Value	
Mobile device usage (Multiple answers)									
Smartphone	No	58	48.7	35	60.3	23	39.7	0.221	a
	Yes	61	51.3	30	49.2	31	50.8		
Feature phone	No	104	87.4	57	54.8	47	45.2	0.915	a
	Yes	15	12.6	8	53.3	7	46.7		
Computer	No	111	93.3	61	55.0	50	45.0	1.000	b
	Yes	8	6.7	4	50.0	4	50.0		
Tablet device	No	113	95.0	63	55.8	50	44.2	0.409	b
	Yes	6	5.0	2	33.3	4	66.7		
Group home phone	No	88	73.9	48	54.5	40	45.5	0.978	a
	Yes	31	26.1	17	54.8	14	45.2		
Socialize with people									
Socialize with group home PMHI	No	64	53.8	40	62.5	24	37.5	0.063	a
	Yes	55	46.2	25	45.5	30	54.5		
Socialize with neighbors	No	97	81.5	60	61.9	37	38.1	<0.001	a
	Yes	22	18.5	5	22.7	17	77.3		
Sources of information for determining disaster experience and evacuation									
Experience of being affected by a natural disaster	No	81	68.1	48	59.3	33	40.7	0.138	a
	Yes	38	31.9	17	44.7	21	55.3		
Sources of information when deciding whether to evacuate (Multiple answers)									
TV	No	52	43.7	32	61.5	20	38.5	0.182	a
	Yes	67	56.3	33	49.3	34	50.7		
Radio	No	95	79.8	54	56.8	41	43.2	0.333	a
	Yes	24	20.2	11	45.8	13	54.2		
Smartphone (internet)	No	71	59.7	41	57.7	30	42.3	0.405	a
	Yes	48	40.3	24	50.0	24	50.0		
Feature phone	No	112	94.1	62	55.4	50	44.6	0.700	b
	Yes	7	5.9	3	42.9	4	57.1		
Computer	No	118	99.2	64	54.2	54	45.8	1.000	b
	Yes	1	0.8	1	100.0	0	0.0		
Local Government Staff	No	105	88.2	59	56.2	46	43.8	0.347	a
	Yes	14	11.8	6	42.9	8	57.1		
Group home staff	No	44	37.0	24	54.5	20	45.5	0.990	a
	Yes	75	63.0	41	54.7	34	45.3		
Assumptions about evacuations from a disaster									
My friends would support me if I had to evacuate	No	91	76.5	51	56.0	40	44.0	0.574	a
	Yes	28	23.5	14	50.0	14	50.0		
My family would support me if I had to evacuate	No	71	59.7	39	54.9	32	45.1	0.935	a
	Yes	48	40.3	26	54.2	22	45.8		
My group home staff would support me if I had to evacuate	No	21	17.6	12	57.1	9	42.9	0.798	a
	Yes	98	82.4	53	54.1	45	45.9		
I can imagine living in a public shelter	No	81	68.1	53	65.4	28	34.6	<0.001	a
	Yes	38	31.9	12	31.6	26	68.4		
I want to stay in my room without evacuating	No	41	34.5	20	48.8	21	51.2	0.353	a
	Yes	78	65.5	45	57.7	33	42.3		
I can’t live in a shelter with many people	No	62	52.1	33	53.2	29	46.8	0.750	a
	Yes	57	47.9	32	56.1	25	43.9		
I am concerned about interpersonal relationships at the shelter	No	34	28.6	21	61.8	13	38.2	0.322	a
	Yes	85	71.4	44	51.8	41	48.2		
I am concerned about stigma from others at the shelter	No	46	38.7	27	58.7	19	41.3	0.479	a
	Yes	73	61.3	38	52.1	35	47.9		

a: χ^2^ test; b: Fisher’s exact test.

**Table 3:** Factors related to the willingness of PMHI to disclose their illness to supporters after the evacuation.

Item	Category	OR	95% CI		*p*-Value
			Lower Limit	Upper Limit	
Sex	Female/Male	0.85	0.35	2.05	0.717
Age group	65 years or older/Under 65 years old	0.34	0.12	1.01	0.052
Type of disability	Mental disability/Mental disability and other disabilities	2.69	0.86	8.46	0.091
Experience of being affected by a natural disaster	No/Yes	1.85	0.76	4.51	0.174
I can imagine living in a public shelter	Yes/No	4.50	1.78	11.43	0.002
Socialize with neighbors	Yes/No	5.63	1.74	18.22	0.004

Binomial logistic regression analysis; Cox–Snell R^2^ = 0.232; Nagelkerke R^2^ = 0.311; OR: odds ratio, CI: confidence interval.

## Data Availability

The data presented in this study are available on request from the corresponding author.

## Public Involvement Statement

No public involvement in any aspect of this research.

## Guidelines and Standards Statement

This manuscript was drafted based on the Strengthening the Reporting of Observational Studies in Epidemiology (STROBE) guidelines for cross-sectional studies [50].
